# The termites of Early Eocene Cambay amber, with the earliest record of the Termitidae (Isoptera)

**DOI:** 10.3897/zookeys.148.1797

**Published:** 2011-11-21

**Authors:** Michael S. Engel, David A. Grimaldi, Paul C. Nascimbene, Hukam Singh

**Affiliations:** 1 Division of Entomology (Paleoentomology), Natural History Museum, and Department of Ecology & Evolutionary Biology, 1501 Crestline Drive – Suite 140, University of Kansas, Lawrence, Kansas 66049-2811, USA; 2 Division of Invertebrate Zoology (Entomology), American Museum of Natural History, Central Park West at 79th Street, New York, New York 10024-5192, USA; 3 Birbal Sahni Institute of Paleobotany, 53 University Road, Lucknow 226 007, India

**Keywords:** India, Tertiary, Eocene, termites, Termitidae, Rhinotermitidae, Stylotermitidae, Neoisoptera

## Abstract

The fauna of termites (Isoptera) preserved in Early Eocene amber from the Cambay Basin (Gujarat, India) are described and figured. Three new genera and four new species are recognized, all of them Neoisoptera – *Parastylotermes krishnai* Engel & Grimaldi, **sp. n.** (Stylotermitidae); *Prostylotermes kamboja* Engel & Grimaldi, **gen. et sp. n.** (Stylotermitidae?); *Zophotermes* Engel, **gen. n.**, with *Zophotermes ashoki* Engel & Singh, **sp. n.** (Rhinotermitidae: Prorhinotermitinae); and *Nanotermes isaacae* Engel & Grimaldi, **gen. et sp. n.** (Termitidae: Termitinae?). Together these species represent the earliest Tertiary records of the Neoisoptera and the oldest definitive record of Termitidae, a family that comprises >75% of the living species of Isoptera. Interestingly, the affinities of the Cambay amber termites are with largely Laurasian lineages, in this regard paralleling relationships seen between the fauna of bees and some flies. Diversity of Neoisoptera in Indian amber may reflect origin of the amber deposit in Dipterocarpaceae forests formed at or near the paleoequator.

## Dedication

It is with great admiration that we dedicate this paper to our dear friend and colleague, Prof. Kumar Krishna, the authority on living and fossil termites. We have had the pleasure of working alongside Kumar for many years now and on numerous projects, none of which would have seen successful completion had it not been for his keen insight and global and encyclopedic knowledge of the Isoptera. Now 83, Kumar continues to be our guide through the wonders and subtle nuances of termite systematics and biology. We look forward to many more years of such pleasurable mentorship and amity.

## Introduction

**Figure 1.**
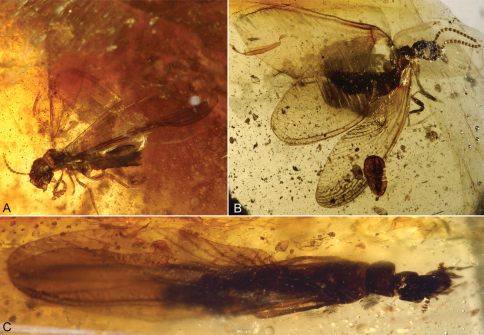
Photomicrographs of Cambay amber (Early Eocene) termites. **A** *Nanotermes isaacae* Engel & Grimaldi, gen. et sp. n., holotype (Termitidae: Tad-262) **B** *Parastylotermes krishnai* Engel & Grimaldi, sp. n., holotype (Stylotermitidae: Tad-277) **C** *Zophotermes ashoki* Engel & Singh, sp. n., holotype (Rhinotermitidae: Tad–42). Not to the same scale.

**Figure 2.**
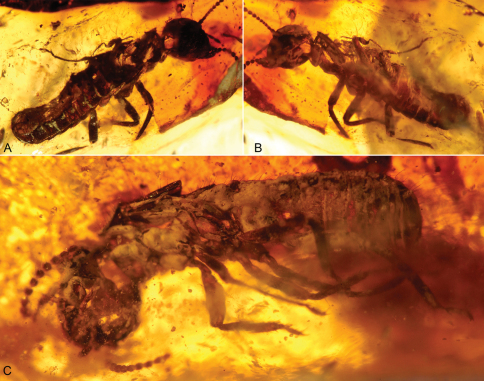
Photomicrographs of dealate male and female of *Prostylotermes kamboja* Engel & Grimaldi, gen. *et* sp. n. (Stylotermitidae: Tad-321C). **A** Dorsal view of female **B** Ventral view of female **C** Ventral view of male. Not to the same scale.

The fossil record of termites has expanded greatly during the last 10–15 years, with numerous new taxa uncovered from deposits throughout the world. Of particular importance are the plethora of new specimens in amber which, with their exceptionally high fidelity of preservation, have permitted dramatic new insights into the history of the order and its paleobiology (e.g., Engel et al. 2009). As revealed in these studies, while termites diversified in the latest Jurassic or earliest Cretaceous into numerous lineages today recognized as the various families and subfamilies (Engel et al. 2009; Ware et al. 2010), as well as several extinct stem groups, they apparently did not rise in abundance or specific diversity until the Tertiary (Engel et al. 2009). Accordingly, the Paleogene record of Isoptera has a special significance, since it provides a window into a major shift in termite evolution, specifically the origin and proliferation of the higher termites in the family Termitidae. Previously the critical windows into these epochs were the Eocene deposits of amber in the Baltic region and Paris Basin (Nel and Bourget 2006; Engel et al. 2007a; Engel 2008). The recent discovery and documentation of abundant amber from western India of Paleogene age is therefore of considerable interest, particularly as termites have been revealed as inclusions (Rust et al. 2010). Herein we provide an overview of the isopteran fauna presently known from the Early Eocene of India (Figs 1, 2), a fauna similar to that of the slightly younger Baltic amber but which most surprisingly harbors the earliest record of Termitidae (Fig. 1A). As excavations and screening of the Indian amber continue, this termite paleofauna will surely grow.

## Material and methods

The Cambay amber deposits, their biotic diversity, and biogeographical significance were reviewed by Rust et al. (2010). Amber was collected by the authors from large lignite mines in Gujarat State, India, during January of 2009, 2010, and 2011. Preparation of pieces followed the methods outlined in Nascimbene and Silverstein (2000). Morphological terminology and the format for the descriptions generally follows that used elsewhere for fossil termites (e.g., Krishna and Grimaldi 2000, 2003, 2009; Wappler and Engel 2003; Engel and Krishna 2007a, 2007b; Engel et al. 2007a, 2007b; Grimaldi et al. 2008; Engel and Gross 2009; Engel and Delclòs 2010). The higher classification adopted herein follows that of Engel et al. (2009). Institutional acronyms are **BSIPL**, Birbal Sahni Institute of Palaeobotany, Lucknow, India; **AMNH**, American Museum of Natural History, New York, and **SEMC**, Snow Entomological Collections, Division of Entomology, University of Kansas Natural History Museum, Lawrence.

## Systematic paleontology

### Family Stylotermitidae Holmgren & Holmgren

#### Genus Parastylotermes

Snyder & Emerson

http://species-id.net/wiki/Parastylotermes

Parastylotermes Snyder & Emerson in Snyder 1949: 378. Type species: *Stylotermes washingtonensis* Snyder, 1931, by original designation.

##### Comments.

The genus *Parastylotermes* was erected by Snyder and Emerson (in Snyder 1949) to accommodate two Tertiary species of Laurasian termites allied to the Recent genus *Stylotermes* Holmgren and Holmgren, from India, Bangladesh, Malaysia, and southern China (Emerson 1971; Krishna et al. in press). Two further species of *Parastylotermes* were subsequently added by Snyder (1955) and Pierce (1958) from Miocene deposits in southern California. Like *Stylotermes*, *Parastylotermes* has trimerous tarsi (a rare condition among the Isoptera) and similar wing pilosity (membrane largely without setae except on scale, where they are numerous and relatively long), in addition to other stylotermitid features (Emerson 1971). *Parastylotermes* differs from *Stylotermes* in the 2-2-2 tibial spur formula (vs. 3-2-2 in *Stylotermes*), M closer to CuA, and more numerously-branched CuA. The hitherto known species were *Parastylotermes robustus* (Rosen) in mid-Eocene Baltic amber (Rosen 1913; Weidner 1955; Emerson 1971; Engel et al. 2007a), *Parastylotermes washingtonensis* (Snyder) from the Miocene Latah Formation of Washington (Snyder 1931), *Parastylotermes frazieri* Snyder from the Miocene of Frazier Mountain, California (Snyder 1955; Emerson 1971), and *Parastylotermes calico* Pierce from the Miocene nodules of the Calico Mountains, California (Pierce 1958; Emerson 1971). Remarkably, a fifth species now has been identified in the Early Eocene Cambay amber, significantly expanding the known distribution of the genus and well into the area today occupied by *Stylotermes*.

#### Parastylotermes krishnai

Engel & Grimaldi sp. n.

urn:lsid:zoobank.org:act:9B0E707D-8F26-4FCE-A806-F708CAFD518D

http://species-id.net/wiki/Parastylotermes_krishnai

Figs 1B 3

##### Holotype.

Imago (sex unknown); Tad-277 (Fig. 1B), India: Gujarat: Tadkeshwar lignite mine, Cambay Formation (Paleo-Eocene), 21°21.400"N, 73°4.532"E, 17–22 January 2010 (BSIPL).

##### Additional material.

**Figure 3.**
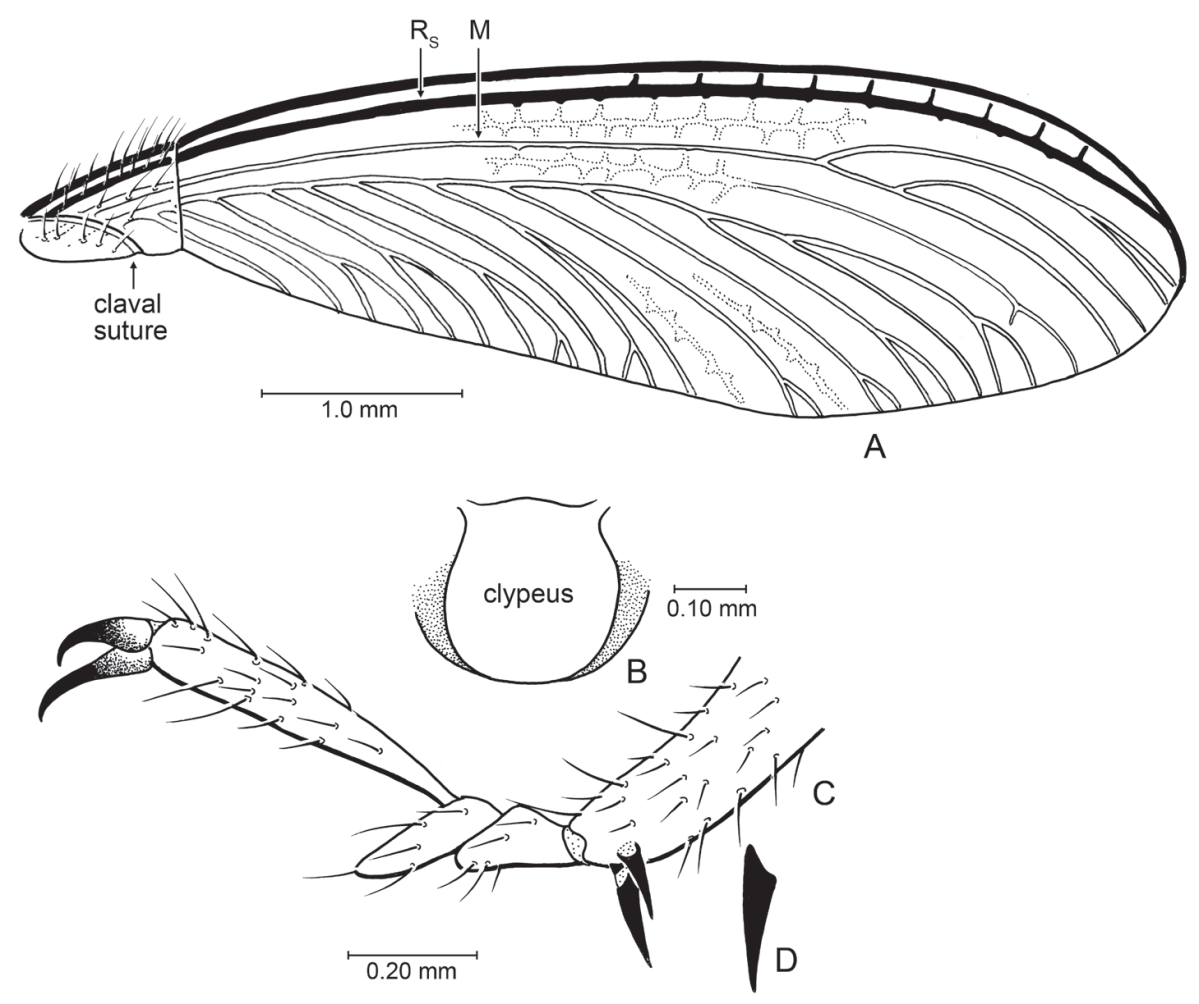
Details of *Parastylotermes krishnai* Engel & Grimaldi, sp. n., holotype (Tad-277). **A** Forewing venation **B** Clypeus (from Tad-96) **C** Pretarsus, tarsus, and extreme apex of tibia **D** Detail of spur.

Imago; Tad-96, India: Gujarat: Tadkeshwar lignite mine, Cambay Formation (Paleo-Eocene), 21°21.400"N, 73°4.532"E, 7–12 January 2009 (AMNH). This specimen is a poorly preserved alate, with much of the specimen crushed and large portions of the upper body, wings, &c. missing. However, the front of the head is not deformed, with a good frontal view of the clypeus (Fig. 3B). The existing wing fragments show a venation very similar to that of the holotype, and the antenna has 14 antennomeres, as in the holotype. These features, along with the trimerous tarsi, strongly suggest that this is an additional individual of this species.

##### Diagnosis.

The new species can be distinguished from all other *Parastylotermes* by the apical branching of the medial vein in the forewing (branching in the apical quarter rather than being unbranched or branched only at the extreme wing apex), less reticulation, more CuA branches (10, versus 7–8 in *Parastylotermes robustus*) and by the smaller number of antennal articles (14 in the new species, 16–17 in *Parastylotermes robustus*, unknown for *Parastylotermes frazieri*, *Parastylotermes washingtonensis*, and *Parastylotermes calico*, which are just forewings preserved as compressions). In all other respects, *Parastylotermes krishnai* matches the description and lectotype (*visum*) for *Parastylotermes robustus* in Baltic amber except for in general metrics and some aspects of coloration (Weidner 1955; Emerson 1971; Engel et al. 2007a).

##### Description.

*Imago*: Total length without wings (as preserved) ~4.0 mm; forewing length 5.7 mm, width 1.7 mm; length of forewing scale 0.8 mm; three maxillary and labial palpomeres. Integument finely imbricate throughout; head dark brown with scattered long, erect, light brown setae, short setae exceptionally sparse; antenna brown, with 14 articles, each with scattered short setae and a few long setae apically; compound eyes round, of moderate size; ocelli not visible owing to preservation of head cuticle; Y-shaped ecdysial cleavage lines and fontanelle not evident (obscured by folding of head cuticle; however, in living Stylotermitidae the fontanelle is exceptionally small and often not visible). Pronotum brown, with scattered, long, fine, erect setae; anterolateral angle acutely rounded, posterior lateral angles broadly rounded, with small medial emargination along anterior border. Legs brown with sparse, short setae except more numerous and stout on tibiae and tarsi; tibial spur formula 2-2-2, perhaps with a single outer spine present on protibia (difficult to discern in holotype), articulating bases of spurs oblique; tarsi trimerous, apical tarsomere longer than combined lengths of basitarsus and second tarsomere, second tarsomere projecting apically beneath base of apical tarsomere; pretarsal claws simple, arolium absent. Forewing scale large, overlapping hind wing scale, humeral margin faintly convex, apical margin straight, CuP (claval fissure) gently arched, meeting posterior margin of scale well before suture, scale with numerous long, erect setae, particularly along humeral margin, without short setae; C and R more darkly pigmented than remaining veins; Sc apparently short, terminating within scale; veins more separated apically than proximally; Rs unbranched, running close and parallel to costal margin, slightly more widely separated from margin apically than proximally; M about midway between R and CuA, branching twice in apical quarter of wing, reaching to wing apex, apical branches of M strongly arched posteriad, such that apices meet wing margin posterior to wing apex; CuA with 10 primary branches reaching to posterior wing margin, apicalmost termination of CuA just posterior to wing apex; veins with sparse, minute setulae; membrane completely bare, between major veins reticulate and with strong, apically-slanting veinlets, particularly midway between R, M, and CuA. Hind wing scale with straight apical margin (suture). Abdomen brown to dark brown; largely crushed and obscured in holotype.

##### Etymology.

The specific epithet is a patronym honoring Prof. Kumar Krishna, world authority on living and fossil termites, in recognition of his many contributions to the subject.

#### Prostylotermes

Engel & Grimaldi gen. n.

urn:lsid:zoobank.org:act:0B6B19C4-F041-4589-A363-8E5D605C6003

http://species-id.net/wiki/Prostylotermes

##### Type species.

*Prostylotermes kamboja* Engel & Grimaldi, sp. n.

##### Diagnosis.

**Figure 4.**
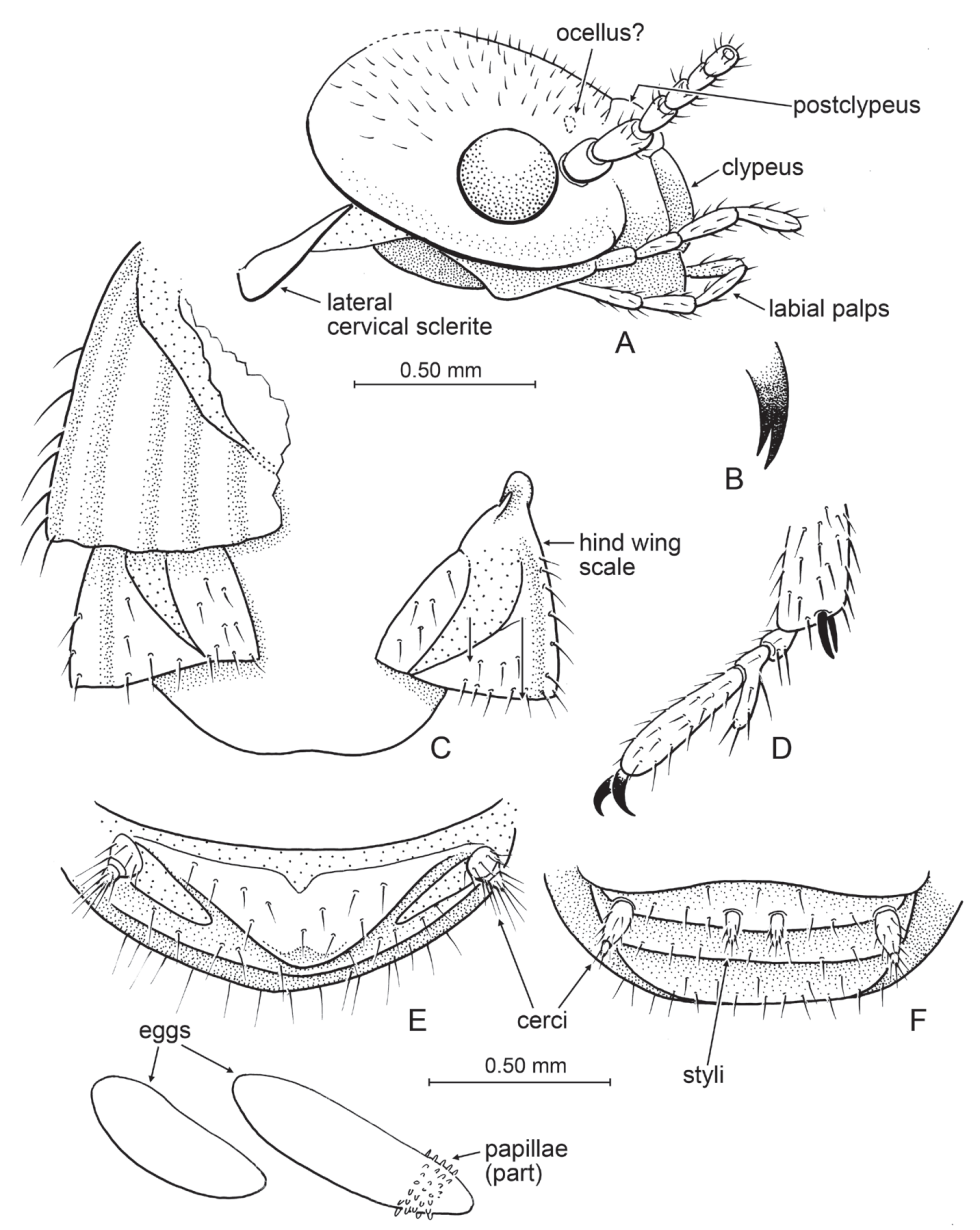
Detail of *Prostylotermes kamboja* Engel & Grimaldi, gen. *et* sp. n. (Tad-321C). **A** Head in lateral aspect **B** Tip of lacinia **C** Dorsal view of wing scales (female specimen) **D** Meso-pretarsus, mesotarsus, and extreme apex of mesotibia (female specimen) **E** Apex of female abdomen, ventral view, with detail of eggs preserved at abdominal apex **F** Apex of male abdomen, ventral view. Scale bars are identical and apply to all figures except the detail enlargements of **B** and **D**.

*Imago*: Head subcircular (Figs 2A, 2B, 4A); compound eyes small, circular; ocelli apparently present, separated from compound eye by more than ocellar diameter (Fig. 4A); postclypeus short, weakly arched (Fig. 4A); antenna with 17 articles. Pronotum flat, narrower than head; tibial spurs 2-2-2; tarsi trimerous, with second tarsomere distinctly projected ventroapically, extension longer than dorsal length of second tarsomere (Fig. 4D). Forewing with scale overlapping base of hind wing scale, slightly larger than hind wing scale (Fig. 4C), scale without numerous setae over surface, with long setae along humeral margin (Fig. 4C) (other stylotermitids have numerous and relatively long setae over the entire scale surface). Cerci short, with two cercomeres (Figs 4E, 4F); styli present in male only, not extending to abdominal apex (Fig. 4F).

##### Etymology.

The new genus-group name is a combination of *pro* (Greek, meaning “before”) and *Stylotermes*, type genus of the family. The name is masculine.

#### Prostylotermes kamboja

Engel & Grimaldi sp. n.

urn:lsid:zoobank.org:act:973DA7E6-C12C-4CB2-8FBB-A61E3CFCEFB9

http://species-id.net/wiki/Prostylotermes_kamboja

Figs 2 4

##### Holotype.

Imago ♀ (dealate) (Figs 2A, 2B); Tad-321C, India: Gujarat: Tadkeshwar lignite mine, Cambay Formation (Paleo-Eocene), 21°21.400"N, 73°4.532"E, 17–22 January 2010 (BSIPL).

##### Paratype.

Imago ♂ (dealate) (Fig. 2C); Tad-321C, same piece and repository as holotype.

##### Diagnosis.

As for the genus (*vide supra*).

##### Description.

*Imago (dealate)*: Total length of female 5.0 mm, of male 3.8 mm; body entirely dark brown, including wing scales and legs, pleural areas lighter. Head of female with length 1.10 mm; compound eye virtually round, diameter 0.25–0.28 mm; fine short pilosity on vertex; postclypeus weakly bulging, length ~0.20 mm, clypeal length ~0.30 mm; fontanelle and coronal ecdysial cleavage line (= Y-shaped suture) not observable as preserved; four maxillary palpomeres, three labial palpomeres; apex of lacinia bifid (Fig. 4B); antenna with 17 articles; flagellomeres slightly and gradually increasing in width distad, basal flagellomere ~0.65x width of apicalmost flagellomere. Pronotum not entirely observable, mostly lost in female and dorsal view obscured in male, portions preserved for female indicate it is narrower than head width. Only wing scales present (wings shed); forewing scale briefly overlapping hind wing scale (by nearly 0.3x length of hind wing scale); both scales with CuP fracture basally very broad, tapered to a point just before or at scale margin; fine setae on costal margin of forewing scale, none on broad surface; some fine setae on broad surface of hind wing scale. Legs with sparse, fine setae on femora and tibiae; tibial spurs 2-2-2, without preapical dorsal spines on tibiae; tarsi trimerous, basitarsomere smallest, second tarsomere with ventroapical extension; distitarsomere 2.5x length of other tarsomeres (excluding second tarsomere extension and pretarsal claws); pretarsal claws simple, arolium absent; meso- and meta- epicoxal regions bulging, slightly explanate. Abdominal tergites and sternites well developed (meeting laterally); abdomen mildly dorsoventrally flattened; apex of abdomen (terminal sternites and tergites) broad, apical margins flattnened; cerci short, with two cercomeres (apicalmost cercomere minute, sometimes separated by distinctive membrane from basal cercomeres [in female]); male with small styli; female without styli.

*Eggs*: Oocytes elliptical, with fine, microscopic chorionic structure; longer one with fine papillae over most of chorion (Fig. 4E). First oocyte length 0.75 mm, width 0.20 mm; second oocyte length 0.53 mm, width 0.20 mm.

##### Etymology.

The specific epithet is treated as a noun in apposition. The name Kamboja (perhaps of Scythian origin) refers to the Indo-Iranian Kshatriya tribe (Hindu warrior elites) who appear in various ancient Indian texts such as the *Vamsa Brahmana* and the *Mahabharata*. In the second century B.C. the Kambojas invaded northern India and took control of various Indo-Arayan territories such as Gujarat, eventually settling the area and lending their name to Khambat (Cambay) and the area in which the amber harboring this species was recovered.

##### Comments.

This piece preserves together two virtually complete dealate adults – one a female, the other a male – though dorsal portions of the female have been lost at the amber surface. Interestingly, two eggs are preserved at the abdominal apex of the female (Fig. 4E).

### Family Rhinotermitidae Froggatt. Subfamily Prorhinotermitinae Quennedey & Deligne

#### Zophotermes

Engel gen. n.

urn:lsid:zoobank.org:act:07D6FDAC-35C5-4DF8-9BD0-0939B19155C7

http://species-id.net/wiki/Zophotermes

##### Type species.

*Zophotermes ashoki* Engel & Singh, sp. n.

##### Diagnosis.

**Figure 5.**
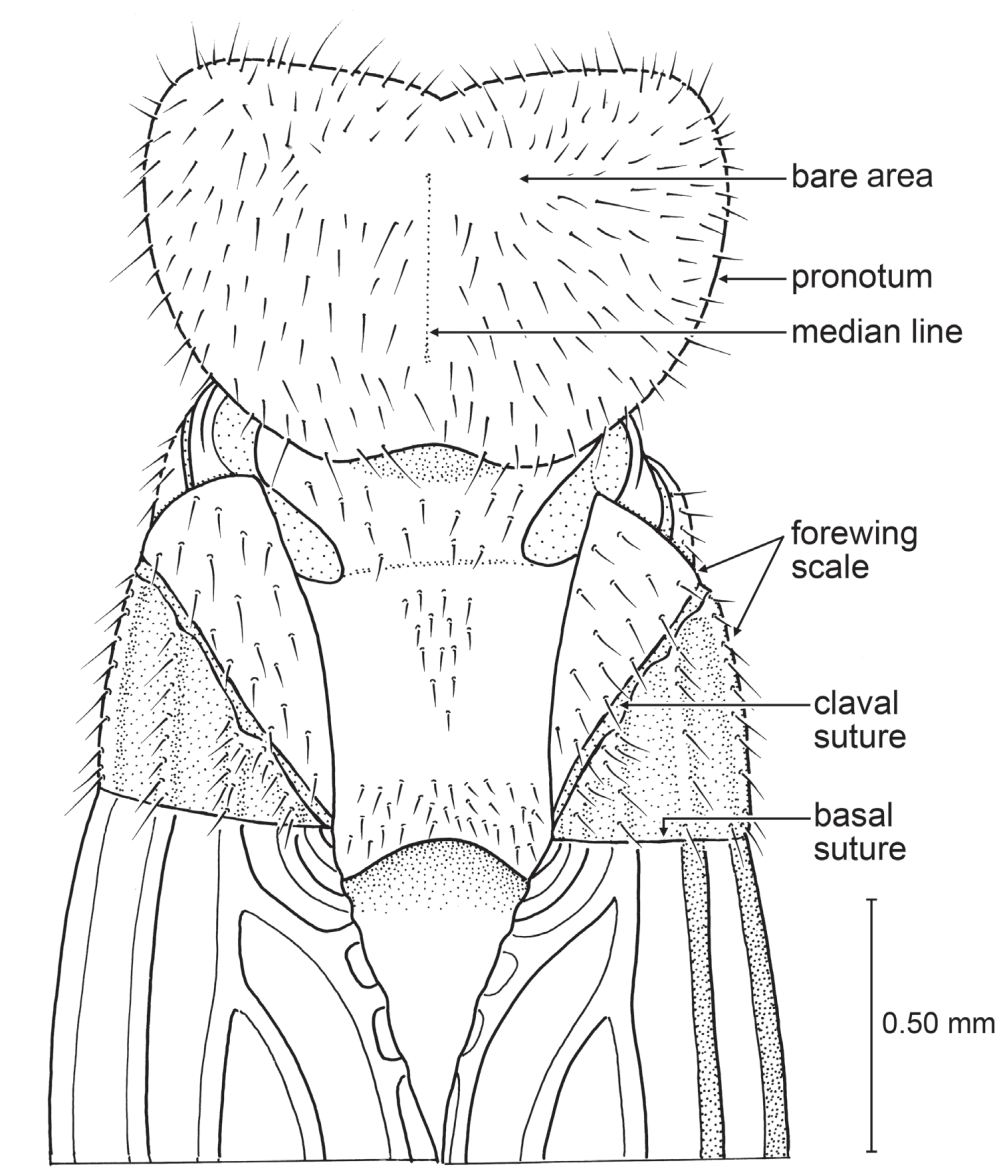
Detail of *Zophotermes ashoki* Engel and Singh, sp. n. (Tad-42), dorsal view of thorax and anterior portion of forewings (slightly reconstructed).

*Imago*: Head not flattened, narrow oval in shape, with sides somewhat parallel (appears similar to condition in Heterotermitinae but there is some compression which may be obscuring slightly roundish borders), posterior margin even; postclypeus without nose-like projection, without groove from fontanelle to apex of labrum, short relative to width, somewhat arched (as in Prorhinotermitinae); compound eyes small; ocelli present. Pronotum flat, narrower than head, anterior margin with medial emargination (Fig. 5); tibial spurs 2-2-2 (3-2-2 in *Prorhinotermes* Silvestri, Psammotermitinae, and Heterotermitinae); tarsi tetramerous. Forewing with scale overlapping hind wing scale; M branching from CuA outside of scale (Fig. 5) (as in Prorhinotermitinae and Psammotermitinae); wing membrane with relatively few setae (as in Prorhinotermitinae).

##### Etymology.

The new genus-group name is a combination of *zophos* (Greek, meaning, “nether world” or “gloom”), and *Termes*, type genus of the Termitidae. The name is masculine.

#### Zophotermes ashoki

Engel & Singh sp. n.

urn:lsid:zoobank.org:act:CA5E2EBA-B8CB-4C11-83E4-B6A5B7E5C1D9

http://species-id.net/wiki/Zophotermes_ashoki

Figs 1C 5 6A

Rhinotermitidae sp.; Rust et al. 2010: 18362–18364, fig. 2G.

##### Holotype.

Imago (sex unknown); Tad-42 (Fig. 1C), India: Gujarat: Tadkeshwar lignite mine, Cambay Formation (Paleo-Eocene), 21°21.400"N, 73°4.532"E, 7–12 January 2009 (BSIPL).

##### Additional material.

Imago (wings only); Tad-97, India: Gujarat: Tadkeshwar lignite mine, Cambay Formation (Paleo-Eocene), 21°21.400"N, 73°4.532"E, 7–12 January 2009 (AMNH).

##### Diagnosis.

As for the genus (*vide supra*).

##### Description.

*Imago*: Total length without wings (as preserved) 4.9 mm; forewing length 6.0 mm; pronotal length (medial) 0.75 mm, width 1.20 mm; length of forewing scale 0.80 mm. Integument of head dark brown, nearly black, except antenna and mouthparts brown; pronotum and remainder of thorax dark reddish brown, legs brown; abdomen dark brown. Integument apparently finely imbricate (where evident). Head relatively large (although left side and much of vertex distorted by compression), length greater than width, lateral borders slightly convex and parallel, with scattered, erect, stout setae, such setae sparse on lateral surface behind compound eye. Compound eyes relatively small, circular, weakly exophthalmic, positioned well anterior on head, separated from posterior border of head by more than compound eye diameter. Fontanelle present, circular, located midway along tangent between middle of compound eyes. Ocelli small, semicircular, positioned anterodorsal to compound eye, separated from compound 2.5–3.0x ocellar diameter. Antenna moniliform, number of articles indeterminate owing to preservation, visible articles with moderately numerous, minute, apically-directed setae and microtrichia. Pronotum slightly wider than long, slightly broader than head, anterior margins slightly conergent mediad, apicolateral corners acutely rounded, lateral margins initially parallel in apical quarter then slightly tapering posteriorly with broadly-rounded posterior corners, medial posterior margin relatively straight; surface with numerous stout, short, suberect, posteriorly-directed setae except those along anterior margins slightly more dense and directed anteriad. Legs with numerous short to moderate-length setae; tibiae without distinct spines; tibial spur formula 2-2-2; tarsi tetramerous; pretarsal ungues simple; arolium absent. Forewing scale large, slightly overlapping hind wing scale; hind wing scale smaller than forewing scale; both fore and hind wing scales covered with numerous, stout, short, erect to suberect setae, such setae intermingled with longer setae, particularly apically along veins; hind wing with C+Sc+R and Rs thick, sclerotized, with ~5 short, perpendicular crossveins connecting them in apical third; M bifurcate in apical half, originating from CuA outside of wing scale; Cu with at least five main branches, two branches with short bifurcate branches at apex; apex of Cu reaches to 0.92x length of wing; no reticulate crossveins present; membrane very finely and densely pimplate. Abdomen apparently with scattered setae similar to those on wing scales.

**Figure 6.**
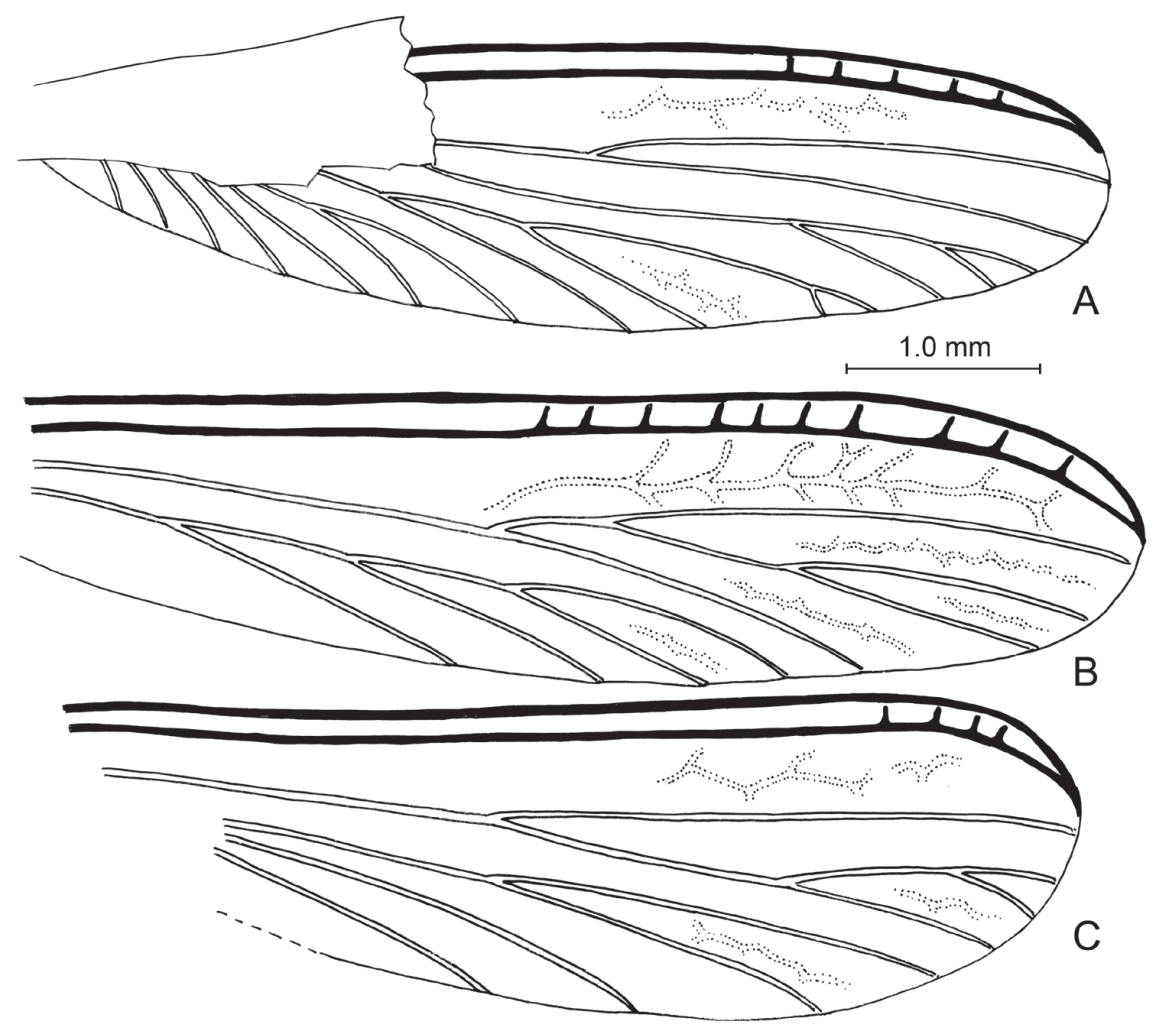
Wing venation of Cambay amber species of *Zophotermes* Engel, gen. n. **A** Hind wing of *Zophotermes ashoki* Engel & Singh, sp. n. (Tad-42) **B** Forewing of *Zophotermes*? sp. (Tad-95) **C** Hind wing of *Zophotermes*? sp. (Tad-278). Scale bar applies to all figures.

##### Etymology.

The specific epithet is a patronym honoring Dr. Ashok Sahni, sage of Indian paleontology and wonderful colleague.

#### Zophotermes ? indeterminate

Figs 6B, 6C

##### Material.

Imago (wing and fragments only); Tad-95, India: Gujarat: Tadkeshwar lignite mine, Cambay Formation (Paleo-Eocene), 21°21.400"N, 73°4.532"E, 7–12 January 2009 (AMNH). Imago (wing fragment only); Tad-278, India: Gujarat: Tadkeshwar lignite mine, Cambay Formation (Paleo-Eocene), 21°21.400'N, 73°4.532'E, 17–22 January 2010 (AMNH). Imago (very badly crushed and obscured); SEMC-F000157, India: Gujarat: Tadkeshwar mine, Surat District, Cambay Basin, 21°19'26"N, 73°4'32"E, 7–12 January 2009 (SEMC). Imago (head, anterior thorax, and wing bases only); Tad-304, India: Gujarat: Tadkeshwar lignite mine, Cambay Formation (Paleo-Eocene), 21°21.400"N, 73°4.532"E, 17–22 January 2010 (AMNH).

##### Comments.

The above four specimens are too poorly preserved to permit conclusive assignment to any particular species but for each the observable details are indicative of a prorhinotermitine and they are apparently of the genus *Zophotermes*. Most, if not all, could be conspecific with *Zophotermes ashoki* described herein but we have hesitated to make a formal assignment as the condition of each is inadequate.

#### Rhinotermitidae indet.

##### Material.

Imago (largely crushed dealate, sex unknown); Tad-155, India: Gujarat: Tadkeshwar lignite mine, Cambay Formation (Paleo-Eocene), 21°21.400"N, 73°4.532"E, 7–12 January 2009 (AMNH).

##### Comments.

This specimen is badly damaged and while it certainly has the appearance of a *Heterotermes*, assignment as to genus, or even subfamily, cannot be made with confidence.

### Family Termitidae Latreille. Subfamily Termitinae? Latreille

#### Nanotermes

Engel & Grimaldi gen. n.

urn:lsid:zoobank.org:act:2D8541FC-A9DD-4593-81B3-405EAE33FBDD

http://species-id.net/wiki/Nanotermes

##### Type species.

*Nanotermes isaacae* Engel & Grimaldi, sp. n.

##### Diagnosis.

*Imago*: Minute termites (ca. 2.0 mm in length excluding wings, forewing ca. 2.6 mm), with head longer than wide and sparsely setose. Labrum possibly without dark, sclerotized transverse band, relatively long; postclypeus prominent and large; fontanelle apparently obscure or obscured (owing to folding of the cuticle); antenna moniliform, with 12 articles, increasing in size apicad (apical flagellomere 2x width of basal flagellomere). Forewing scale small, not overlapping hind wing scale, with basal suture relatively straight, humeral margin straight, all veins originating within scale, CuP relatively straight and terminating before basal suture; wing membrane microtrichose, not reticulate, not infuscate; Sc and R pigmented, remainder of veins faint; M and CuA become nebulous to spectral by about one-third wing length (CuA can be discerned by tilting specimen; apical two-thirds of M cannot be detected). Tibial spur formula 2-2-2; tibiae without outer spines; tarsi trimerous (similar in this respect to *Indotermes* Roonwal and Sen-Sarma of the Apicotermitinae: *Speculitermes* Group); pretarsal claws simple, arolium absent. Pronotum wider than long, slightly narrower than head; anterior margin straight, with very faint medial notch, apicolateral corners acutely rounded; lateral borders parallel-sided, with broadly-rounded posterior corners; posterior border relatively straight; setae nearly absent except a few along margins.

##### Etymology.

The new genus-group name is a combination of *nanos* (Gr., meaning, “small”), as this is probably the smallest known alate termite, and *Termes*, type genus of the Termitidae. The name is masculine.

#### Nanotermes isaacae

Engel & Grimaldi sp. n.

urn:lsid:zoobank.org:act:8A3AF869-D486-4A22-94A5-C8F2DDC66D5D

http://species-id.net/wiki/Nanotermes_isaacae

Figs 1A 7

##### Holotype.

Imago; Tad-262 (Fig. 1A), India: Gujarat: Tadkeshwar lignite mine, Cambay Formation (Paleo-Eocene), 21°21.400"N, 73°4.532"E, 17–22 January 2010 (BSIPL).

##### Diagnosis.

As for the genus (*vide supra*).

##### Description.

As described for the genus with the following details: *Imago*: Total length without wings (as preserved) 2.0 mm; forewing length 2.60 mm; head length ~0.40 mm; length of head to base of clypeus 0.30 mm; clypeal medial length 0.06 mm; pronotal length (medial) 0.18 mm, width ~0.275 mm; length of forewing scale 0.15 mm. Integument of head and abdomen generally dark brown except labrum, postclypeus, and pronotum brown, antennae and legs light brown, labrum with apical margin white (as in some Nasutitermitinae). Integument (where evident) faintly imbricate to smooth. Head largely without setae except for a few laterally; forewing scale with only some sparse short setae.

**Figure 7.**
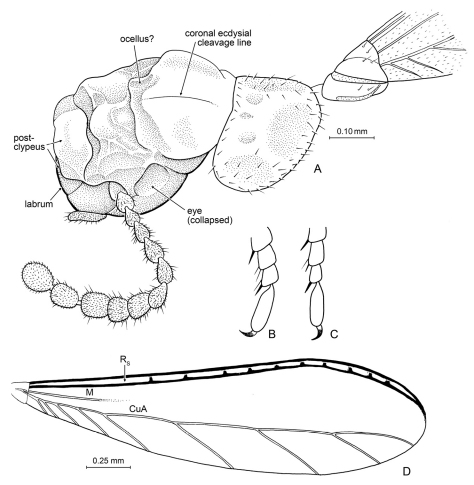
Details of *Nanotermes isaacae* Engel & Grimaldi, gen. et sp. n. (Tad-262). **A** Head (as preserved, showing preservational distortion), pronotum, and base of right forewing **B** Protarsus, pro-pretarsus, and extreme apex of protibia **C** Metatarsus, meta-pretarsus, and extreme apex of metatibia **D** Forewing (reconstructed from both wings). Detail enlargements in **B** and **C** not to same scale.

##### Etymology.

The specific epithet is a matronym for Ms. Charlotte Isaac, who diligently processed and screened amber, and who discovered the holotype among many other interesting inclusions.

## Discussion

Although exploration of Cambay amber remains in its initial stages it is remarkable that such an interesting diversity of termites has already been recovered, with representatives of three different families, including the earliest evidence of the Termitidae. This relative diversity of Neoisoptera may reflect the origin of the amber deposit in dipterocarp forests formed at or near the paleoequator. The species here documented show largely Laurasian connections, particularly with the mid-Eocene fauna of Baltic amber, a pattern mirrored for many other insect lineages (e.g., bees, *Pareuthychaeta* [Diptera, Diastatidae]: Engel unpubl. data; Grimaldi and Singh in press). For example, *Parastylotermes* is largely distributed in western North America and northern Europe. Another interesting connection is highlighted by *Zophotermes*, which has affinities to *Prorhinotermes*. The 11 species of *Prorhinotermes* are largely insular, occurring widely on tropical islands in the Old and New World except for the coasts of Central America, southern Florida, and the Cape York Peninsula (Emerson 1952; Gay and Barrett 1983). It is fascinating to note that a prorhinotermitine-like lineage would be found on the Indian subcontinent just prior to complete suturing with Asia.

*Parastylotermes krishnai* can be readily distinguished from the other species of the genus owing to the more deeply branched medial vein in the forewing and the smaller number of antennal articles. A new genus could have been established for the species but this is presently unwarranted as the observed differences are relatively minor and, while putatively apomorphic, might render *Parastylotermes* paraphyletic. It could of course also be asked whether *Parastylotermes* is simply a stem group to *Stylotermes* and already paraphyletic with respect to the latter. While no phylogenetic analysis yet exists for the species of Stylotermitidae it would appear on the surface that *Parastylotermes* is monophyletic, or more precisely that at least *Parastylotermes robustus* and *Parastylotermes krishnai* are related. The fossil species from western North America are fragmentary, and their relationships are obscure. Most basal Neoisoptera have a 3-2-2 tibial spur formula and this is likely the plesiomorphic condition for this group, suggesting that the more reduced condition observed in *Parastylotermes*, i.e., 2-2-2 (loss of one of the protibial spurs), is synapomorphic for the genus, or perhaps between *Parastylotermes* and *Prostylotermes*, the latter discussed below.

*Prostylotermes kamboja* is a fascinating discovery in that it exhibits many putative plesiomorphic traits for the entire Neoisoptera clade, symplesiomorphies such as the presence of two cercomeres, styli in the male (absent in *Parastylotermes* and *Stylotermes*: Emerson 1971), and a forewing scale that briefly overlaps the hind wing scale (also present in *Parastylotermes*, forewing scale just meets hind wing scale in *Stylotermes*). However, the species simultaneously has the trimerous tarsal condition of stylotermitids, a feature so distinctive among these primitive neoisopterans. Between the two genera of Stylotermitidae, *Prostylotermes* shares the 2-2-2 tibial spur formula with *Parastylotermes* and, if this is derived within the family as discussed above, could represent a synapomorphy for these two genera. Alternatively, the 2-2-2 condition is primitive for Stylotermitidae, with modern *Stylotermes* exhibiting a reversal to the putatively primitive condition (3-2-2) for Neoisoptera. *Prostylotermes* is remarkable in that it partially “deconstructs” the stylotermitid diagnosis, in plesiomorphic features only, relative to basal Neoisoptera.

*Zophotermes ashoki* is particularly fascinating in that it agrees with Prorhinotermitinae in all traits except for the 2-2-2 tibial spur formula. It shares with Prorhinotermitinae and Psammotermitinae the unique branching of the forewing M from CuA outside of the scale (Fig. 5), otherwise unknown among the Rhinotermitidae. It lacks the flattened head or other distinctive traits of Psammotermitinae (*vide supra*). As noted above, the distribution of the 3-2-2 tibial spur formula across Rhinotermitidae, and even Neoisoptera as a whole, suggests that it is a plesiomorphic condition for these subfamilies, in which case the 2-2-2 tibial formula in *Zophotermes* is apomorphic. It is tantalizing to speculate that *Zophotermes ashoki* lacked a true worker caste as is unique to *Prorhinotermes* among modern Rhinotermitidae.

While it is difficult to identify phylogenetic affinities of *Nanotermes* given the challenge of working solely from alates, the significance of this fossil is that it is the oldest definitive representative of the Termitidae. All previous records of Termitidae are from the Late Oligocene or younger (Nel and Paicheler 1993; Martins-Neto and Pesenti 2006; Krishna and Grimaldi 2009). *Nanotermes* thereby extends the age of this family by a further 20 million years. Given that Termitidae comprise today more than 75% of all termite species and that the family as a whole likely originated in the Paleocene, it is remarkable that this overwhelming diversity came about during the mid-Tertiary, such that relatively modern faunas were established by the earliest Miocene (Krishna and Grimaldi 2009). Termitids apparently were rare in the Paleogene and their rise in abundance and ecological dominance likely was rapid sometime after the Early Oligocene (Engel et al. 2009). Accordingly, it would not be surprising to find Eocene or Early Oligocene termitids which cannot be confidently assigned to any particular subfamily. Indeed, subfamilial placement of *Nanotermes* is challenging and it could represent a stem-group termitid. However, given the paucity of observable features its enigmatic nature may reflect absence of data rather than truly plesiomorphic or mosaic combinations of characters removing it from modern subfamilial lineages. Regardless, *Nanotermes* can be excluded from the Macrotermitinae owing to the absence of a dark, sclerotized transverse band on the labrum and from the Termitinae owing to the 2-2-2 tibial spur formula. The dorsal surface of the head is not ideally preserved but there does not appear to be a slit-like fontanelle, and the structure may be somewhat obscured. If this is the case, then the condition would be reminiscent of Apicotermitinae. The 2-2-2 tibial spur formula is found among the Apicotermitinae, Termitinae, Syntermitinae (other taxa in these subfamilies have the plesiomorphic condition of 3-2-2 which is universal in Macrotermitinae, Foraminitermitinae, Sphaerotermitinae, and Cubitermitinae), and universally in the Nasutitermitinae. Among the subfamilies in which the 2-2-2 condition may be found only the *Amitermes* Group (Termitinae, formerly Amitermitinae) and Nasutitermitinae have the reduced number of antennal articles as low as that observed for *Nanotermes*, although some Syntermitinae come close with 13 antennal articles. Syntermitinae have a relatively large fontanelle which, despite the distortion to the head, would presumably be apparent. Similarly, there does not appear to be a slit-like fontanelle present on *Nanotermes* either, which would, in combination with a postclypeus that is arched and relatively long (length about one-half width) seems to exclude an assignment to Nasutitermitinae. It seems most likely that *Nanotermes* is a termitine and perhaps allied to the *Amitermes* group of genera, although definitive attribution must remain for the time being uncertain. Certainly the trimerous tarsi are unique among all of these lineages but are perhaps not surprising for such a diminutive species.

## Supplementary Material

XML Treatment for Parastylotermes

XML Treatment for Parastylotermes krishnai

XML Treatment for Prostylotermes

XML Treatment for Prostylotermes kamboja

XML Treatment for Zophotermes

XML Treatment for Zophotermes ashoki

XML Treatment for Zophotermes

XML Treatment for Rhinotermitidae

XML Treatment for Nanotermes

XML Treatment for Nanotermes isaacae
